# Prediction of antimicrobial resistance from MALDI-TOF mass spectra using machine learning: a validation study

**DOI:** 10.1128/jcm.01186-25

**Published:** 2025-11-26

**Authors:** Niklas Wiesmann, Dominic Enders, Antje Westendorf, Raphael Koch, Frieder Schaumburg

**Affiliations:** 1 Institute of Medical Microbiology, University of Münster 9185, Münster, Germany; 2 Institute of Biostatistics and Clinical Research, University of Münster 9185, Münster, Germany; 3 Institute of Medical Informatics, University of Münster 9185, Münster, Germany; Endeavor Health, Evanston, Illinois, USA

**Keywords:** artificial intelligence, machine learning, drug resistance, microbial, anti-bacterial agents, spectrometry, mass, matrix-assisted laser desorption-ionization

## Abstract

Matrix-assisted laser desorption-ionization-time of flight (MALDI-TOF) mass spectra can be used to predict antimicrobial resistance (AMR) using machine learning (ML). This study aimed to validate the performance of ML models for AMR prediction using own and publicly available MALDI-TOF data and to test how these models perform over time. Mass spectra of *Escherichia coli* (*n* = 7,897), *Klebsiella pneumoniae* (*n* = 2,444), and *Staphylococcus aureus* (*n* = 4,664) from routine diagnostics (Germany) and the DRIAMS-A database (Switzerland) were used. Six classification models were benchmarked for AMR prediction using cross-validation (regularized logistic regressions [LR], multilayer perceptrons [MLP], support vector machines [SVM], random forests [RF], gradient boosting machines [LGBM, XGB]). Performance was prospectively observed for 18 months after training. The performance of AMR prediction evaluated by the mean area under the receiver operating characteristic curve (AUROC) was comparable between the DRIAMS-A data set and own data. The best predictive performance (classifier, AUROC) on own data was achieved for oxacillin resistance in *S. aureus* (RF, 0.85), ciprofloxacin resistance in *E. coli* (XGB, 0.83), and piperacillin-tazobactam resistance in *K. pneumoniae* (XGB, 0.81). ML performance was poor if training and test data were unrelated in terms of location and time. Performance (change in AUROC) decreased within 18 months after training for *S. aureus* (oxacillin resistance, RF: −0.10), *E. coli* (ciprofloxacin, XGB: −0.19), and *K. pneumoniae* (piperacillin-tazobactam, XGB: −0.25). The performance of ML for the prediction of AMR based on MALDI-TOF data is good (AUROC ≥ 0.8) but classifiers need to be trained on local data and retrained regularly to maintain the performance level.

**IMPORTANCE:**

MALDI-TOF mass spectrometry can be used not only for bacterial species identification but also for the prediction of antimicrobial resistance (AMR) using machine learning (ML). Such an approach would provide antimicrobial susceptibility test results one day earlier than conventional routine diagnostics. This is essential for an early targeted treatment to reduce mortality of severe infections. We show that the performance of ML for the prediction of AMR based on MALDI-TOF data is good (AUROC ≥ 0.8). However, the ML models need to be trained on local data and retrained regularly to maintain a good performance.

## INTRODUCTION

The early start of an effective antimicrobial treatment is key to reduce mortality of patients with severe infections (1–3). While species identification has been significantly accelerated in recent years (e.g., genome-based detection of pathogens, ultra-short incubation before MALDI-TOF mass spectrometry), there is still an unmet medical need for rapid antimicrobial susceptibility testing (AST) (4). Artificial intelligence, in general, and machine learning (ML), in particular, offer promising approaches for the prediction of antimicrobial resistance (AMR). The majority of current studies focus on prediction of AMR using whole genome sequencing (WGS), which still has a turn-around time of hours to days and is not applicable in many routine diagnostic settings (5, 6). In contrast, data from MALDI-TOF mass spectrometry might be more appropriate as these data are affordable and available within minutes. Moreover, many laboratories already use MALDI-TOF mass spectrometry for bacterial species identification. Using these data also for AMR prediction would provide not only the species of the tested organism but also AMR at the same time.

MALDI-TOF mass spectra can be used already for the prediction of methicillin resistance in *Staphylococcus aureus* (7) or vancomycin resistance in *Enterococcus faecium* (8). This approach was further optimized by Weis et al. to predict a broad range of AMR in clinically important species using ML (9). The performance of these models (displayed as the area under the receiver operating characteristic [AUROC] curve) can be good for the prediction of ciprofloxacin resistance in *Escherichia coli* (0.76), cefepime resistance in *Klebsiella pneumoniae* (0.76), ceftazidime-avibactam resistance in *Pseudomonas aeruginosa* (0.87), or oxacillin resistance in *S. aureus* (0.80) (9, 10). It is currently unclear whether these prediction models are generally valid or if they need to be adjusted to the location and the time of data collection. Various confounders might impact on the performance of prediction (e.g., devices from different manufacturers, maintenance of mass spectrometer, clonal characteristics, and evolution of circulating isolates in one region). The aim of this study was to assess whether MALDI-TOF mass spectrometry data can be used to predict AMR if training and test data are completely independent in terms of location and time. Therefore, the objective was to perform a validation of ML models using own MALDI-TOF spectra and external spectra that are geographically and timely unrelated.

Furthermore, we wanted to determine prospectively whether the prediction performance changes depending on the age of the training data set.

## MATERIALS AND METHODS

### Isolate collection

We set up a database of MALDI-TOF mass spectra with corresponding AMR-profiles in the same way as the publicly available “Database of Resistance Information on Antimicrobials and MALDI-TOF Mass Spectra A” (DRIAMS-A, University Hospital Basel, Switzerland, 2015–2018) (9). Our data were collected from routine diagnostics at the University Hospital Münster, Germany (01/2023–12/2024), and encompass approximately 27,000 samples across various pathogens. Samples were obtained from all patients (in- and outpatients).

**Fig 1:**
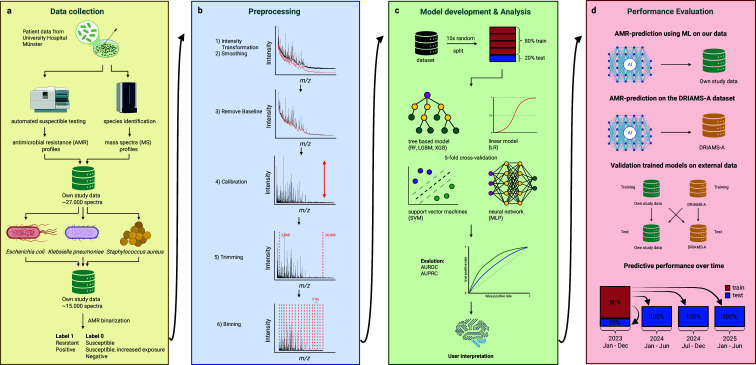
Data workflow. Datasets were created from MALDI-TOF mass spectra (MS) and antimicrobial susceptibility test (AST) profiles (**a**). A total of ~27,000 spectra were reduced to ~15,000 by selecting three species (*Escherichia coli*, *Klebsiella pneumoniae,* and *Staphylococcus aureus*). Accumulated data were binarized. A six-step preprocessing pipeline was established and resulted in 6,000 feature vectors (**b**). For model development, hyperparameter optimization, and performance evaluation, we applied 10 random train-test splits (80%/20%) with inner 5-fold cross-validation on the training set (**c**). We trained random forests (RF), light gradient boosting machines (LGBM), eXtreme Gradient Boosting (XGB), regularized logistic regressions (LR), support vector machines (SVM), and multilayer perceptrons (MLP). Performance metrics were area under the receiver operating characteristic curve (AUROC) and area under the precision-recall curve (AUPRC). We further performed a validation on external data and assessed predictive performance of ML over time (**d**). Abbreviations: DRIAMS-A, database of resistance information on antimicrobials and MALDI-TOF mass spectra.

We included all available *S. aureus*, *E. coli,* and *K. pneumoniae*, comprising both screening swabs (colonization isolates) and clinical specimens from infections (infection isolates, Table 1). Samples with incomplete data (species identification, AST) were excluded *post hoc* (Fig. 1a).

**TABLE 1:** Overview of species-antimicrobial combinations used for the prediction of antimicrobial resistance and corresponding resistance rates (Germany, 2023–2024)

Species	Colonization/ infection (%/%)	Antimicrobial agent	Samples (*n*)	Patient cases (*n*)	Patients (*n*)	Resistance rate (% of samples [*n*])
*Escherichia coli*	15.5%/84.5%	Ampicillin	7,873	5,940	4,829	52.1% (4,100)
		Ciprofloxacin	7,897	5,954	4,841	17.6% (1,391)
		Cefotaxime	7,879	5,942	4,833	15.8% (1,247)
		Trimethoprim-sulfamethoxazole	7,885	5,952	4,838	28.0% (2,208)
*Klebsiella pneumoniae*	26.5%/73.5%	Ciprofloxacin	2,444	1,641	1,402	12.9% (316)
		Cefotaxime	2,442	1,640	1,401	16.3% (398)
		Trimethoprim-sulfamethoxazole	2,442	1,639	1,400	14.7% (360)
		Piperacillin-tazobactam	2,427	1,633	1,397	29.7% (722)
*Staphylococcus aureus*	29.2%/70.8%	Inducible clindamycin resistance	4,519	3,552	2,833	15.6% (704)
		Oxacillin	4,648	3,646	2,894	10.8% (501)
		Benzylpenicillin	4,664	3,663	2,908	61.7% (2,880)

### Species identification and antimicrobial susceptibility testing

MALDI-TOF mass spectrometry was performed with the MALDI Biotyper sirius one System (Bruker Daltonics, Bremen, Germany) using different versions of flexControl software depending on the time of analysis (3.4.207.20, 3.4.207.48, and 3.4.207.59). For the identification of each species, we used the Biotyper Database (BDAL V11/12/2023). Mass spectrometry profiles were saved in the original fid format of the manufacturer.

AST was performed with Vitek2 automated system (bioMérieux, Marcy-l'Étoile, France) and AST cards P654 (*S. aureus*), N371 and N432 (Enterobacterales from urine), or N214 and N428 (Enterobacterales from all other specimen). AST results were interpreted using the European Committee on Antimicrobial Susceptibility Testing (EUCAST) clinical breakpoints in the current versions of the respective test years (11, 12). AST results were categorized in binary labels as “resistant” and “susceptible/susceptible, increased exposure.”

### Data matching

Mass spectra and AST profiles were matched based on the combination of the internal identifiers (order, case, patient, isolate). If MALDI-TOF was repeated for species identification (e.g., due to an unreliable identification score), the most recent measurement was used. Similarly, if AST was repeated for one isolate (e.g., due to incomplete or inconsistent results), the latest measurement was included in the final data set.

### Feature extraction

To ensure comparability, own mass spectra were preprocessed in the same way as the DRIAMS-A data (i.e., intensity transformation, smoothing, baseline removal, total ion count normalization, trimming to the range of 2.000–20.000 *m*/*z*, partition into 3 *m*/*z* bins) resulting in 6,000 features (9). Processing of MALDI-TOF mass spectra was done using “R” and the package *“MALDIquant*” (13) (Fig. 1b).

### Selection of ML models

To assess the performance of ML models for AMR-prediction, we trained and tested various types of ML models separately (Table S1 and S2). For that purpose, *E. coli*, *K. pneumoniae,* and *S. aureus* were selected as they are among the major clinically relevant species in our setting and predominant in the DRIAMS-A data set (9). In our data set, antimicrobials were only selected if AMR rates were ≥10% (Table 1), to ensure a reasonable proportion of resistant isolates needed for model training. We applied the same ML algorithms to the DRIAMS-A data set and the three main species-antimicrobial combinations as reported by Weis et al. (9).

The ML training set up was a sequence of 10 experiments each using random splits with 80% of the data for training and 20% for testing (9). For each experiment, we performed a fivefold cross-validation on the training set using grid search to determine the optimal hyperparameter configuration for each learner. We used the same tuning spaces as Weis et al. (9) (Table S1). The best model was then trained again on all training data and evaluated against the test data of the respective experiment. This approach is similar to a nested 10 × 5 cross-validation. During training, samples were weighted to adjust for class imbalances. All resamplings were constructed using stratification by resistance profile to ensure similar resistance rates across all subsequent training and test folds. Moreover, the resamplings were constructed to account for a clustered data structure (i.e., multiple isolates per patient case).

We studied the general performance of six classification models (regularized logistic regressions [LR], random forests [RF], light gradient-boosting machines [LGBM], eXtreme Gradient Boosting [XGB], multilayer perceptrons [MLP], support vector machines [SVM], Fig. 1; Table S2).

All ML algorithms were implemented using “R” (v4.2.1) (14) and the mlr3 framework (15). The benchmark was built upon the packages *“mlr3tuning,” “mlr3pipeline*s,” and *“mlr3resampling”* (Table S1).

### Evaluation metrics

Predictive performance was calculated as the mean area under the receiver operating characteristic curve (AUROC) across all 10 experiments which plots sensitivity against (1 − specificity). While this measure is independent of the class ratio (i.e., resistance rate), it enables comparisons between the performances of different species-antimicrobial combinations. The AUROC reflects how well a classifier can distinguish resistant from susceptible isolates across all possible decision thresholds. An AUROC of 0.5 indicates random guessing, whereas a value of 1.0 indicates perfect separation. We used the following interpretation for AUROC: ≥0.9 “very good, or excellent”; 0.8–<0.9 “good”; 0.7–<0.8 “moderate, fair, or acceptable”; 0.6–<0.7 “poor, weak, or low”; 0.5–<0.6 “failed or random” (16).

We also report the area under the precision–recall curve (AUPRC) which plots the positive predictive value against the sensitivity of a classifier to predict resistance. Unlike AUROC, AUPRC depends on the positive class ratio (i.e., the proportion of resistant isolates). It highlights the classifier’s ability to correctly identify resistant isolates among all those predicted as resistant. In practical terms, a high AUPRC means that if the model flags an isolate as resistant, it is likely to be truly resistant—a property that becomes crucial in clinical settings.

For the interpretation of our trained ML models, we performed a SHAP (SHapley Additive exPlanations) analysis to identify those features with the highest contribution to model predictions. This concept uses classical Shapley values, which originate from coalitional game theory, and allows the interpretation of model output contributions for each feature (17). We restricted our analysis to tree-based models, for which the computation is feasible using the TreeSHAP algorithm (18, 19).

## RESULTS

### AMR-prediction using ML on our data

We included mass spectra of *E. coli* (*n* = 7,897), *K. pneumoniae* (*n* = 2,444), and *S. aureus* (*n* = 4,664) in the final data set for the prediction of 11 antimicrobial resistances (Table 1). The most frequent specimens were from urine (*n* = 5,951, 39.7%), followed by respiratory tract (*n* = 3,349, 22.3%) and gastrointestinal tract (*n* = 2,025, 13.5%, Table S3).

**Fig 2:**
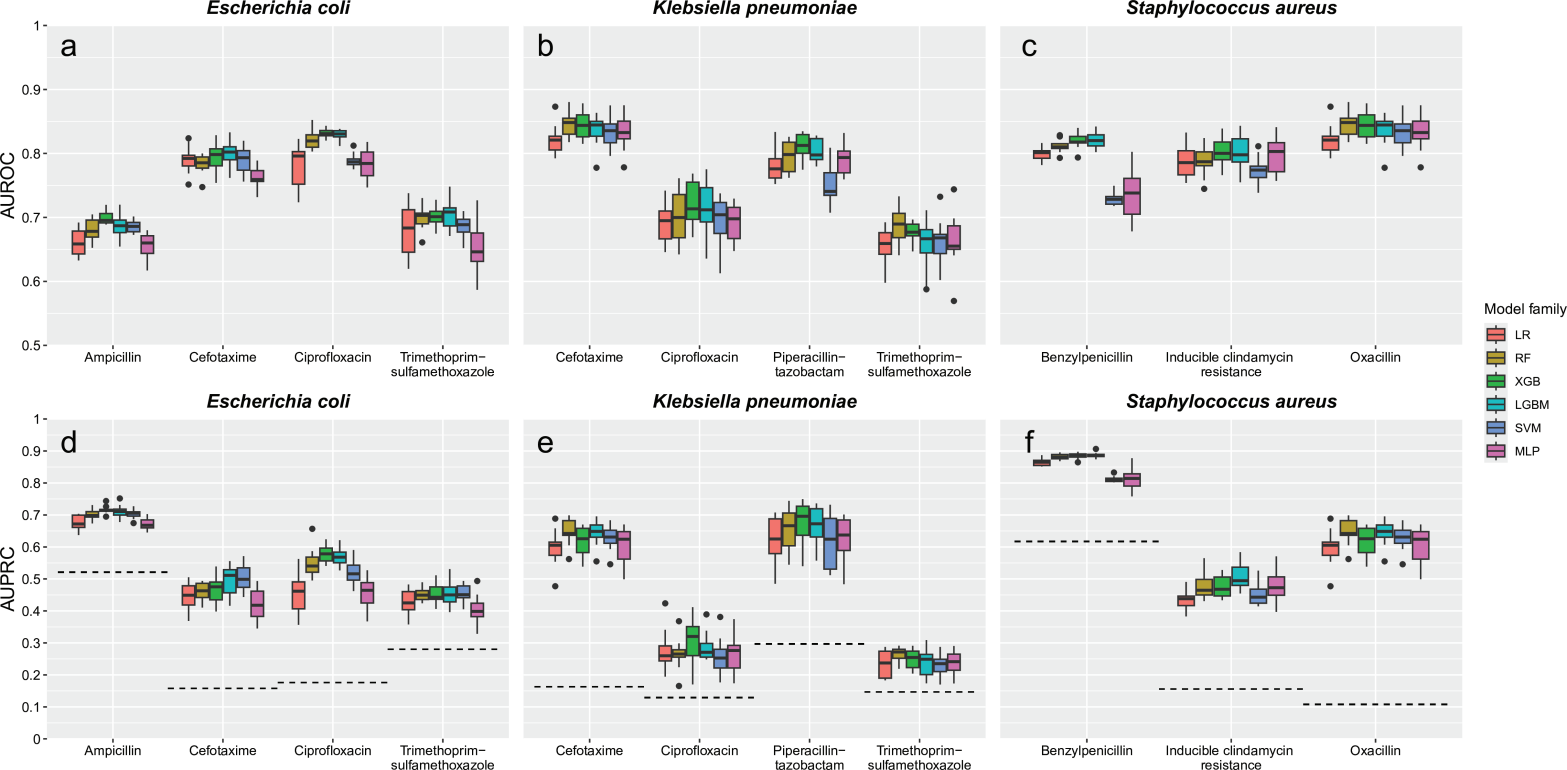
Boxplots of benchmark results for tuned ML-models using six different learners grouped by species and antimicrobial agent. Performance is reported as the area under the receiver operator characteristic curve (AUROC, **a–c**) and the area under the precision-recall curve (AUPRC, **d–f**) on the test data for 10 random train/test splits. Models were tuned using a grid search and fivefold cross-validation. The dashed lines (**d–f**) indicate the resistance rates of the respective antimicrobial agent as a lower bound for the AUPRC. Dots represent outliers. Abbreviations: LR, logistic regression; RF, random forest; XGB, eXtreme Gradient Boosting; LGBM, Light Gradient-Boosting Machine; SVM, support vector machine; MLP, multilayer perceptron

While there are marked differences in performance between different species-antimicrobial combinations, the different models showed comparable results within each combination (Fig. 2). The prediction of ciprofloxacin resistance (AUROC) among the classifiers depended on the species and was better for *E. coli* (0.78–0.83) than for *K. pneumoniae* (0.69–0.72). In contrast, the prediction of cefotaxime and trimethoprim-sulfamethoxazole was comparable for *E. coli* and *K. pneumoniae* (Fig. 2). For *S. aureus*, the performance of AMR prediction ranged between 0.73 and 0.85 (Fig. 2).

For all 11 combinations, the best performing model was always tree-based (RF, XGB, or LGBM). Performance (classifier, AUROC) was best for the prediction of oxacillin resistance in *S. aureus* (RF, 0.85 ± 0.02), for ciprofloxacin-resistance in *E. coli* (XGB, 0.83 ± 0.01), and for piperacillin-tazobactam resistance in *K. pneumoniae* (XGB, 0.81 ± 0.02).

The results of the SHAP analysis for selected models are displayed in Fig. S1. Distributions of the most influential features for the presented models are spread across the entire range of the mass spectra. Feature importance is mainly uniformly distributed among the top features, except for the random forest model predicting oxacillin resistance in *S. aureus* which has only a few highly important features.

### AMR-prediction on the DRIAMS-A data set

When training our ML algorithms on the DRIAMS-A data set, we obtained models with nearly identical performance as reported by Weis et al. (9) (Table 2). Best performing models (AUROC) were LGBM for the prediction of ceftriaxone resistance in *E. coli* (0.75 ± 0.04), XGB for ceftriaxone resistance in *K. pneumoniae* (0.75 ± 0.04), and MLP for oxacillin resistance in *S. aureus* (0.82 ± 0.04).

**TABLE 2:** Comparison of the best performing machine learning models from different studies trained on DRIAMS-A data (9)^*a*^

Species	Predicted antimicrobial resistance	Reference for the developed machine learning models	Best model	AUROC ± SD	AUPRC ± SD
*Escherichia coli*	Ceftriaxone/cefotaxime	This study	LGBM	0.75 ± 0.04	0.29 ± 0.05
		Weis et al. (9)	LGBM	0.74 ± 0.02	0.30 ± 0.03
*Klebsiella pneumoniae*	Ceftriaxone/cefotaxime	This study	XGB	0.75 ± 0.04	0.31 ± 0.06
		Weis et al. (9)	MLP	0.74 ± 0.04	0.33 ± 0.07
*Staphylococcus aureus*	Oxacillin	This study	MLP	0.82 ± 0.04	0.46 ± 0.07
		Weis et al. (9)	LGBM	0.80 ± 0.03	0.49 ± 0.06

^*a*^ AUROC, area under the receiver operating characteristic curve; AUPRC, area under the precision-recall curve; SD, standard deviation; XGB, eXtreme Gradient Boosting; LGBM, Light Gradient-Boosting Machine; MLP, multilayer perceptron.

### Cross-site validation of trained models on external data

**Fig 3:**
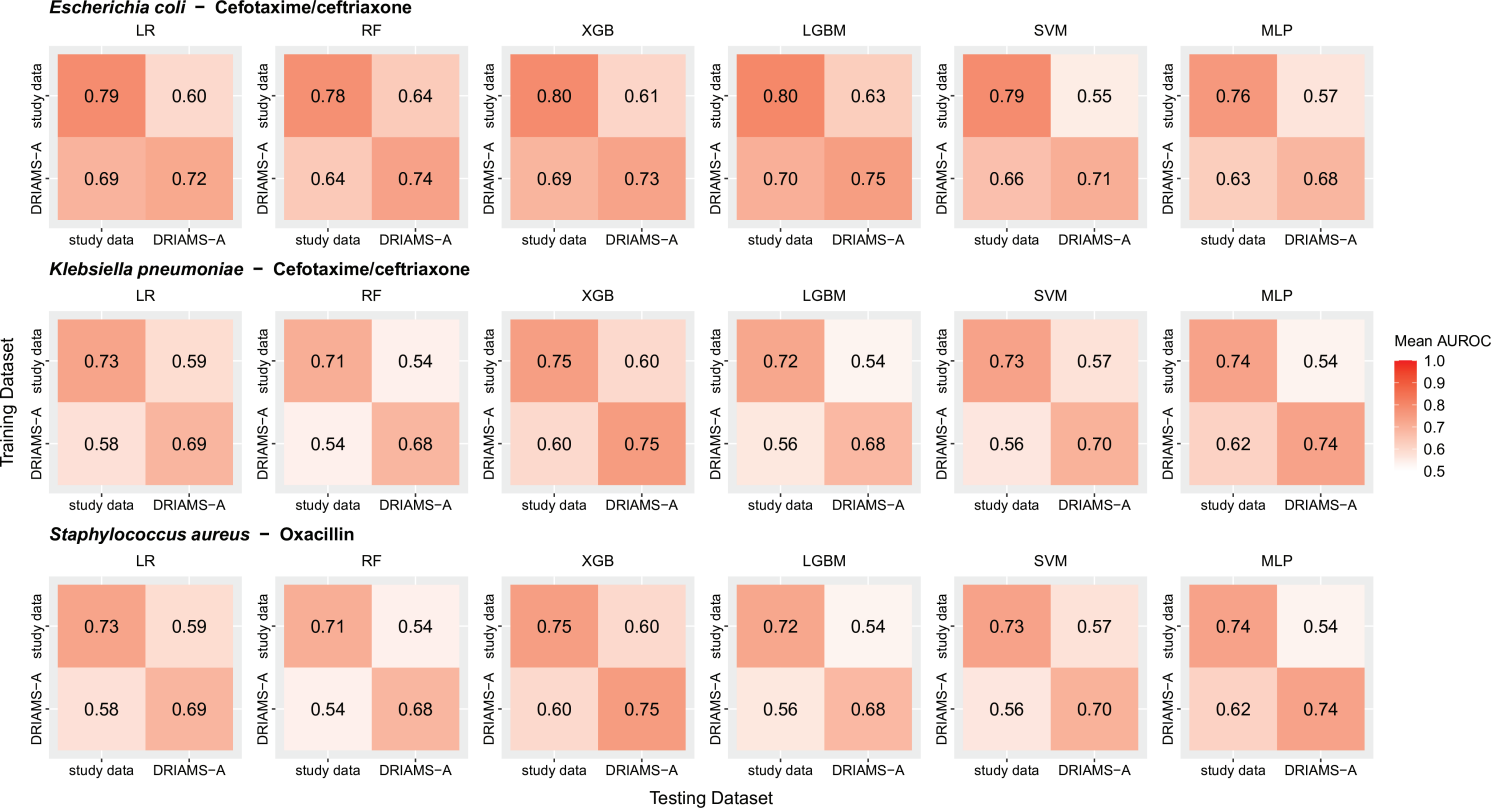
Cross-site validation of ML models. Heat maps are a visual representation of the performance of various learners when trained on one dataset (our own study data or DRIAMS-A) and evaluated on the respective other data set. The data sets were unrelated in terms of location and time (DRIAMS-A: Switzerland, 2015–2018; own study data: Germany, 2023–2025). Performance is displayed as the area under the receiver operating characteristic curve (AUROC, mean over best models for 10 random train-test split) for each combination of learners and species-antimicrobial combination from Weis et al. (9). Abbreviations: LR, logistic regression; RF, random forest; XGB, eXtreme Gradient Boosting; LGBM, Light Gradient-Boosting Machine; SVM, support vector machine; MLP, multilayer perceptron; DRIAMS-A, Database of Resistance Information on Antimicrobials and MALDI-TOF mass spectra—site A (9).

To evaluate the applicability of our ML models to external data and *vice versa*, we compared performances when using different combinations of training and test data (our own data vs. DRIAMS-A, Fig. 1d and 3). Weis et al. used ceftriaxone and we used cefotaxime as a label for resistance against 3rd generation cephalosporins in *E. coli* and *K. pneumoniae*. Since potential resistance mechanisms against the two compounds are identical, we consider merging these compounds into one label (ceftriaxone/cefotaxime) to be valid. We evaluated the performance of the models for the prediction of ceftriaxone/cefotaxime resistance in *E. coli* and *K. pneumoniae* and oxacillin-resistance in *S. aureus*. For the two datasets (DRIAMS-A, own study data), we tested all four combinations of training and test data sets (e.g., models were trained on DRIAMS-A and tested on our data, Fig. 3). We observed a striking drop in performance when the data sets used for training and testing differed. For instance, AUROC dropped between 0.065 and 0.225 (range) across all species-antimicrobial and learner combinations when DRIAMS-A data were used for training and prediction was performed on own data, or vice versa (Fig. 3). All these changes in AUROC were statistically noticeable (*P* < 0.05 for all comparisons using Wilcoxon signed-rank tests on the paired AUROCs for each mode from the 10 experiments).

### Predictive performance over time

For clinical application, it is important to know to what extent the time between the collection of the training and test sets has an influence on performance eventually making regular updates (i.e., retraining of classifiers) necessary (Fig. 1d). Access to recent training samples improves AMR-prediction performance (9). Here, we assessed how a trained classifier performs over time after being trained once at the beginning of the prospective observation period. We restricted our model training to data from Jan 2023 to Dec 2023 and evaluated the prediction performance of the trained classifiers on prospective data from three six-month periods (i.e., Jan 2024–Jun 2024, Jul 2024–Dec 2024, Jan 2025–Jun 2025, Fig. 4; Fig. S2).

**Fig 4:**
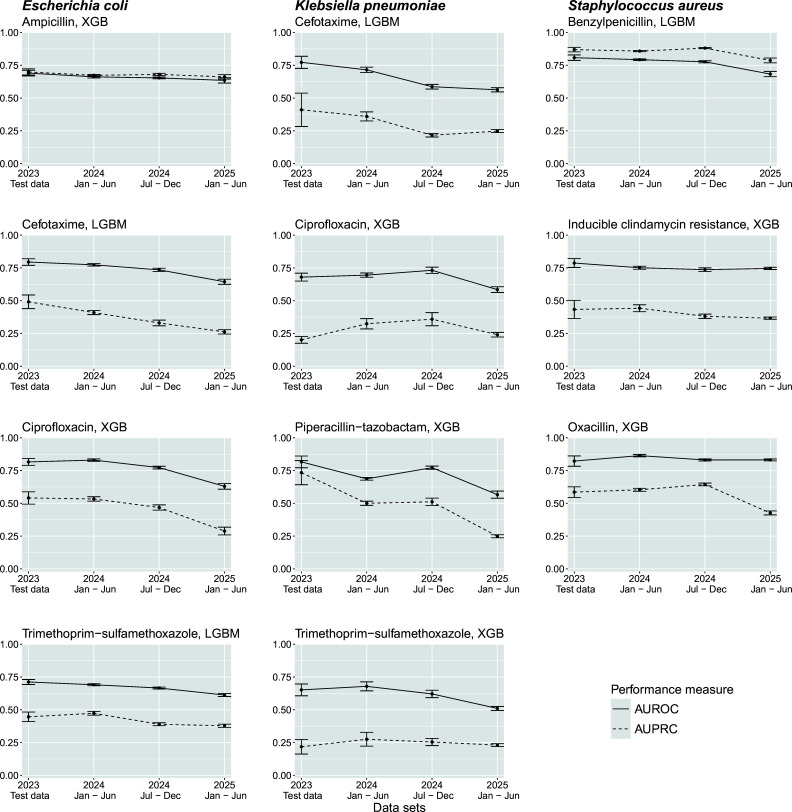
Performance of trained classifiers over time. Own study data were used for each species-antimicrobial combination, models are trained on data from Jan 2023 to Dec 2023. The model with the best AUROC performance in 2023 was then evaluated on prospective data from the following three half years. Model performance (averaged over best models from 10 random train-test splits, mean ± SD) in terms of area under the receiver operating characteristic curve (AUROC, solid lines) and area under the precision-recall curve (AUPRC, dashed lines). Abbreviations: XGB, eXtreme Gradient Boosting; LGBM, Light Gradient-Boosting Machine.

For most species-antimicrobial combinations, we found a steady decline in predictive performance (Fig. S2). Performance decreased (expressed in reduction of AUROC) within 18 months after training for *S. aureus* (oxacillin resistance, RF: −0.10), *E. coli* (ciprofloxacin, XGB: −0.19), and *K. pneumoniae* (piperacillin-tazobactam, XGB: −0.25). This temporal trend was consistent across almost all model families and can be observed with regard to both AUROC and AUPRC. Noteworthy, most resistance rates remained essentially constant over the observation period (Table S4 and Fig. S3) with the exception of resistance against piperacillin-tazobactam and trimethoprim-sulfamethoxazole (+7.5% and −13.6%, respectively) in *K. pneumoniae*.

## DISCUSSION

We assessed the performance of ML models on the prediction of AMR using MALDI-TOF mass spectra. Main finding is an overall good performance of tree-based ML models if they were trained and tested on data that is related in terms of location and time. If training and test data differ in the site of specimen collection, the performance decreases. The predictive performance decreases as well if the time gap between training data and test data increases.

Apart from two classifiers (SVM, MLP), the prediction of AMR in *S. aureus* using own data was comparable among the different models (Fig. 2). In contrast, the predictive power varied considerably for *E. coli* and *K. pneumoniae* depending on the tested antimicrobial agent (Fig. 2) which is in line with observations made on the DRIAMS-A data set (9). Our trained models performed slightly better than the ones reported by Weis et al. for the same species-antimicrobial combinations (9). Since the model selection and training setup were purposely identical (except for different software implementations), the increased performance is most likely due to the larger sample size and higher positive class ratio in our data set.

In our data set, the prediction of cefotaxime resistance outperforms trimethoprim-sulfamethoxazole resistance both in *E. coli* and *K. pneumoniae* (Fig. 2). Cefotaxime resistance is mostly conferred by extended-spectrum beta-lactamases of the CTX-M type with CTX-M-15 being the predominant worldwide (20). With regard to protein structure (the relevant substrate for MALID-TOF), this class is homogeneous, as the different CTX-M subtypes only differ in point mutations. Mechanism of trimethoprim-sulfamethoxazole resistance is more heterogeneous (i.e., efflux pumps, regulatory changes, and mutations of *dhfr* and/or *dhps* [inlc. *fol*P, *sul*I, *sul*II]) (21). The performance is usually better (but less generalizable) in a homogeneous than in a heterogeneous data set. Previous studies have shown that incorporating the site of specimen collection into heterogeneous data sets improves model performance (22).

In general, the predictive performance of ML on MALDI-TOF data is promising but still not acceptable as an alternative for AST in routine diagnostics. Whether the predictive performance of classifiers can be improved by adding patient or environmental data, optimizing the processing of MALDI-TOF (e.g., dynamic binning [10]), hyperparameter optimization, deep learning, or the use of advanced neural network architectures is currently investigated by us.

We trained our ML models on the DRIAMS-A data set and found almost identical performance metrics as reported by Weis et al. (Table 2) (9). This validates our replication of their ML setup and confirms the reproducibility of their findings.

We tested the performance of the ML models if training and test data were unrelated in terms of location (Switzerland vs Germany) and time (2015–2018 vs 2023–2025).

The performance was poorer if training and test data were not from the same location/time. Most notably, this effect can be observed throughout all tested combinations of species, antimicrobials, and models, suggesting systematic differences between mass spectra, AST, and/or AMR from these two datasets (e.g., due to different devices, settings, procedures, software, AST methods and interpretation guidelines, class imbalances, data heterogeneity). DRIAMS-A did not only use AST results from VITEK2 automated systems but also from other sources (e.g., disk diffusion, gradient diffusion) (9). DRIAMS-A AST results were interpreted using EUCAST clinical breakpoints, but at the time of collection, “I” was defined as “intermediate” and grouped with resistant isolates after binary labeling, while in our data set, “I” is defined as “susceptible, increased exposure” and grouped with susceptible isolates. The different definition of the category “I” does not explain the poorer performance because this discrepancy is relevant for only two of the three species-antimicrobial combinations (i.e., cefotaxime in *E. coli* and *K. pneumoniae*) and affects less than 1% of samples (Table S5).

A poorer performance of the classifiers if training and test data were unrelated (time, location) is in line with the study from Switzerland where the same observation was already made if data sets were from different sites within the same country (9).

To some extent, our findings might be explained by underlying biological differences in the pathogens from different geographical areas. Since the two datasets were collected more than five years apart, it is possible that there have been changes in the epidemiology of the pathogens (e.g., changing resistance rates) (23). The observed lack of transferability suggests that classifiers should be trained using local data or that further development of high-performing models should be pursued using pooled data from different sites. In both cases, this highlights the importance of systematic and standardized collection and preparation of mass spectrometry and antimicrobial resistance data.

The prospective assessment of predictive performance revealed that AUROC already decreases within 6–12 months which was most pronounced for *K. pneumoniae* (cefotaxime, piperacillin-tazobactam, Fig. 4; Fig. S2). This effect might be due to changes in pathogen characteristics over time. Technical modifications in data collection (e.g., recalibration of devices, software updates) probably have a little effect on the decline of performance as bacterial test standards are usually applied for each sample run for calibration and fine-tuning purposes. In either case, our observations demonstrate that prediction models should be retrained regularly. As a consequence, for implementation in clinical practice, mass spectrometry and antimicrobial resistance data should be collected continuously so that deployed prediction models can regularly be validated and updated in case their performance starts to deteriorate.

In an ideal scenario, a reinforcement learning framework should be implemented in which models are continuously updated using available reference results (e.g., Vitek data available 24 h later). A more pragmatic solution, supported by the findings of this study, is to schedule retraining on a bi-annual basis to ensure sustained model performance in routine practice (Fig. 4; Fig. S2). Noteworthy, maintenance of ML models, including retraining, requires both personnel expenses and expertise.

Our study has limitations. First, we used a simple grid search for the hyperparameter optimization with the parameter space from Weis et al. (9) to enable a direct model comparison. Therefore, we did not apply advanced hyperparameter optimization techniques, such as hyperbands or Bayesian optimization, to more detailed tuning spaces (24). This could have resulted in better-performing ML models. Second, the mass spectrometry covered a range between 2,000 and 20,000 Da (i.e., range that is used for species identification in routine diagnostics), which excludes distinct proteins that confer resistance (e.g., PBP2a in oxacillin resistance with a molecular weight of 76,000 Da). A broader spectrum might have improved the performance of the ML models. Furthermore, the models do not necessarily select features that are directly associated with AMR mechanisms. Instead, the models decide which parts of the data they consider most suitable for predicting resistance. Thus, it is unclear if the features highlighted by the SHAP analysis are directly related or only correlated to AMR mechanisms. Similarly, Shapley values are known to be potentially misleading for highly correlated features. Whether the features used by the models can be explained biologically is a question for further research.

### Conclusion

The performance of ML for the prediction of AMR based on MALDI-TOF data is good. However, classifiers need to be trained on local data and retrained regularly to maintain the performance level.

## Data Availability

Our data are not publicly available as they contain patient-related identifiers to match antimicrobial susceptibility test results and MALDI-TOF Mass spectrometry data and therefore fall under the data protection act. However, selected data and code can be made available upon request.

## SUPPLEMENTAL MATERIAL

Supplemental Material jcm.01186-25-s0001.pdf Tables S1 to S5; Figures S1 to S3.

ASM does not own the copyrights to Supplemental Material that may be linked to, or accessed through, an article. The authors have granted ASM a non-exclusive, world-wide license to publish the Supplemental Material files. Please contact the corresponding author directly for reuse.
