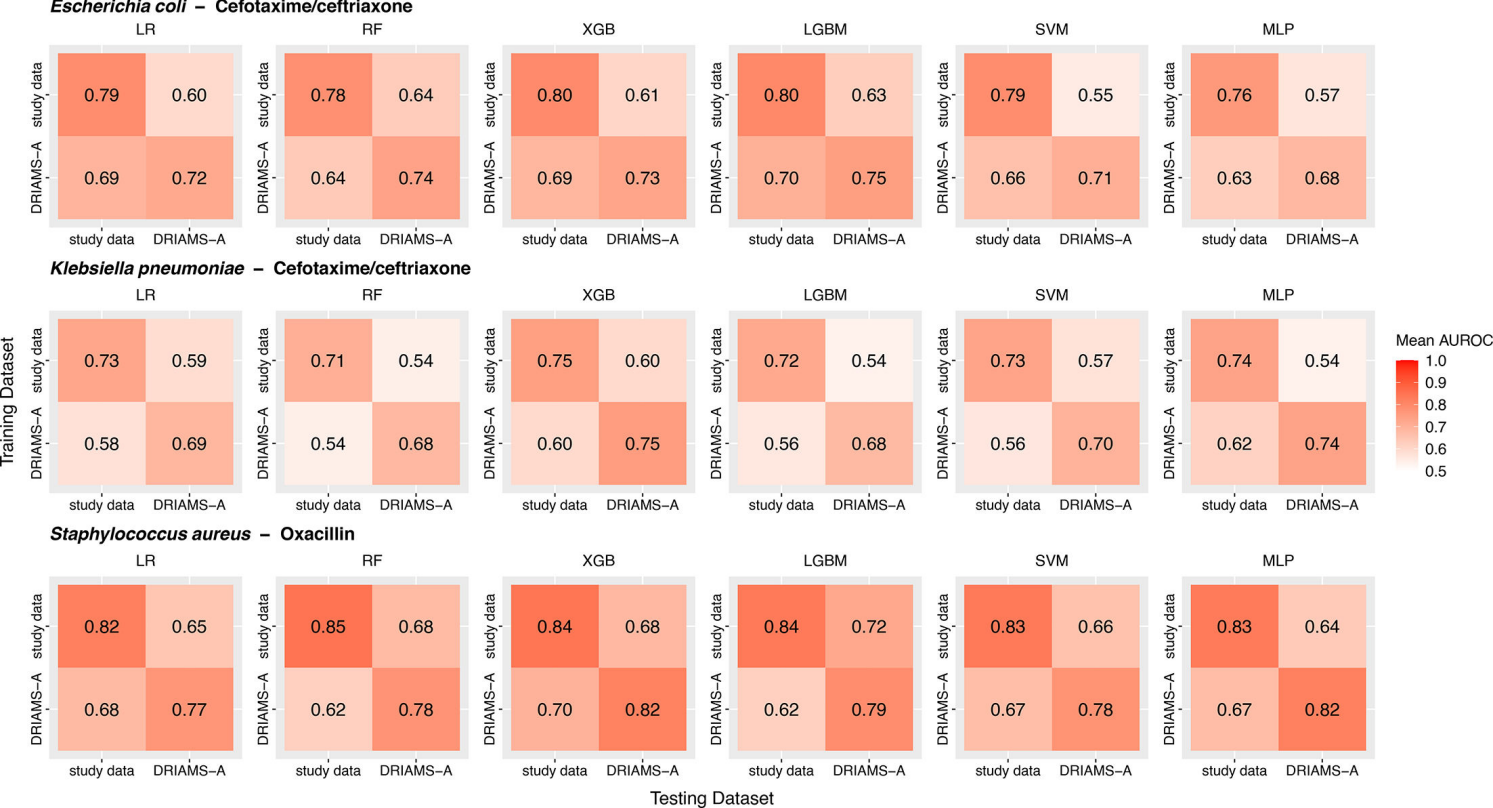# Correction for Wiesmann et al., “Prediction of antimicrobial resistance from MALDI-TOF mass spectra using machine learning: a validation study”

**DOI:** 10.1128/jcm.01884-25

**Published:** 2026-02-13

**Authors:** Niklas Wiesmann, Dominic Enders, Antje Westendorf, Raphael Koch, Frieder Schaumburg

## AUTHOR CORRECTION

Volume 63, no. 12, e01186-25, 2025, https://doi.org/10.1128/jcm.01186-25. Figures 2 and 3 should appear as shown in this correction.

In Fig. 2b and 2e, the boxplots for the *Klebsiella pneumoniae*−Cefotaxime combination were duplicates of the boxplots for *Staphylococcus aureus*−Oxacillin (Fig. 2c and 2f) and were therefore incorrect. The correct boxplots are now displayed for the *Klebsiella pneumoniae*–Cefotaxime combination.

In Fig. 3, we mistakenly overrode the results for the *Staphylococcus aureus*−Oxacillin combination with the graphics and values from *Klebsiella pneumoniae*−Cefotaxime/Ceftriaxone. This is now corrected.

The results stated in the tables and body of the article are correct and the conclusions remain intact. We apologize for these errors, which did not affect the results or conclusions.

**Fig 2:**
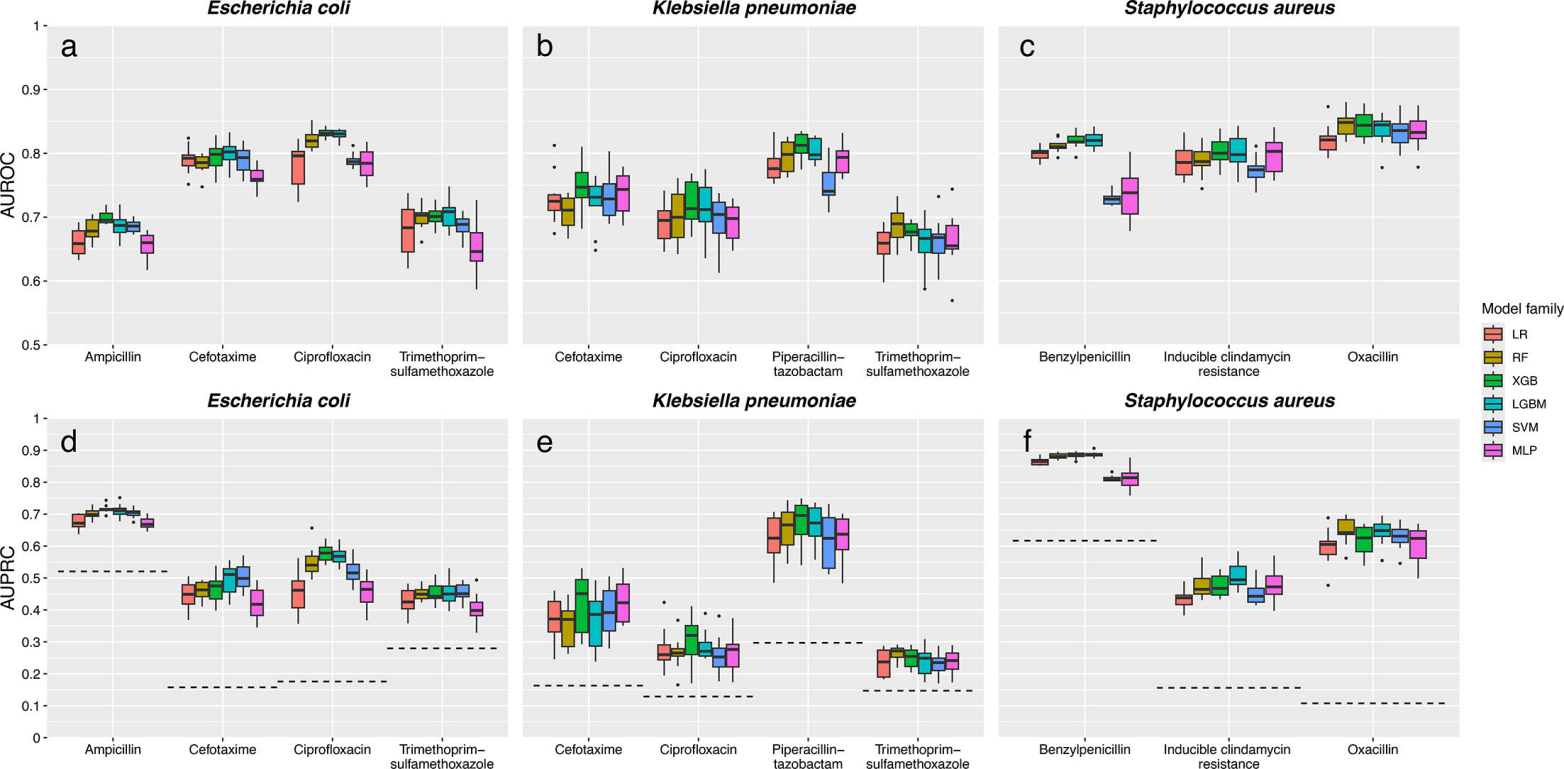


**Fig 3:**