# Supplementary material for: Prediction of antimicrobial resistance from MALDI-TOF mass spectra using machine learning: a validation study
Source: J Clin Microbiol. 2025 Nov 26;63(12):e01186-25. doi: 10.1128/jcm.01186-25 (PMC12710331; doi:10.1128/jcm.01186-25)
Supplement: Supplemental Material — Tables S1 to S5; Figures S1 to S3. [file jcm.01186-25-s0001.pdf]

Supplemental material

Table S1: Learners and R-packages used for model training

Learner	R-package, version	Tuning grid space	Link	Ref.
LR - logistic regression	glmnet, v4.1-8	standardize = TRUE, FALSE<br>s = 10^(-3),…,10^3<br>alpha = 0,1	https://doi.org/10.32614/CRAN.package.glmnet	(1)
RF - random forest	ranger, v0.17.0	standardize = TRUE, FALSE<br>num.trees = 25,50,100,200<br>mtry = n_features, sqrt(n_features), log2(n_features)<br>splitrule = gini, extratrees, hellinger<br>replace =TRUE, FALSE	https://doi.org/10.32614/CRAN.package.ranger	(2)
XGB - extreme gradient boosting	xgboost, v1.7.8.1	standardize = TRUE, FALSE<br>booster = gbtree, gblinear<br>eta = 10^(-4),…,10^0	https://doi.org/10.32614/CRAN.package.xgboost	(3)
LGBM - light gradient boosting machine	lightgbm, v3.3.0	standardize = TRUE, FALSE<br>boosting = gbdt, rf<br>data_sample_strategy = bagging, goss<br>learning_rate = 10^(-3),…,10^3<br>num_iterations = 25,50,100,200	https://doi.org/10.32614/CRAN.package.lightgbm	(4)
SVM - support vector machine	e1071, v1.7-16	standardize = TRUE, FALSE<br>cost = 10^(-3),…,10^3<br>kernel = linear, radial<br>gamma = 1/n_features	https://doi.org/10.32614/CRAN.package.e1071	(5)
MLP - multilayer perceptron	mlr3torch, v0.2.1	standardize = TRUE, FALSE<br>neurons = (512,256,128), (512,128,64), (256,64), (256,128)	https://github.com/mlr-org/mlr3torch	(6)
**Further packages used**	future, v1.58.0		https://doi.org/10.32614/CRAN.package.future	(7)
	mlr3, v0.23.		https://doi.org/10.32614/CRAN.package.mlr3	(8)
	mlr3extralearners, v1.0.0		https://github.com/mlr-org/mlr3extralearners	(9)
	mlr3pipelines, v0.8.0		https://doi.org/10.32614/CRAN.package.mlr3pipelines	(10)
	mlr3resampling, v2024.4.14		https://doi.org/10.32614/CRAN.package.mlr3resampling	(11)
	mlr3tuning, v1.4.0		https://doi.org/10.32614/CRAN.package.mlr3tuning	(12)
	mlr3verse, v0.3.1		https://doi.org/10.32614/CRAN.package.mlr3verse	(13)
	treeshap, v0.3.1		https://doi.org/10.32614/CRAN.package.treeshap	(14)
	shapviz, v0.10.2		https://doi.org/10.32614/CRAN.package.shapviz	(15)

The tuning spaces for hyperparameter optimization were chosen exactly as in (16) to enable a fair comparison between our models and those in (16). Abbreviation: ref., reference

Table S2: Predictive performance for selected antimicrobial resistances of different learners

Species	Antimicrobial agent	Performance measure	Learner					
			Logistic regression	Random forest	Extreme Gradient Boosting	Light Gradient-Boosting Machine	Support vector machine	Multilayer perceptron
Escherichia coli	Ampicillin	AUROC	0.661 ± 0.021	0.681 ± 0.018	**0.699 ± 0.011**	0.688 ± 0.019	0.685 ± 0.009	0.655 ± 0.022
		AUPRC	0.675 ± 0.025	0.700 ± 0.018	**0.716 ± 0.013**	0.711 ± 0.021	0.703 ± 0.014	0.672 ± 0.019
	Ciprofloxacin	AUROC	0.780 ± 0.036	0.823 ± 0.017	**0.832 ± 0.007**	0.829 ± 0.009	0.789 ± 0.011	0.783 ± 0.025
		AUPRC	0.455 ± 0.063	0.549 ± 0.048	**0.577 ± 0.027**	0.569 ± 0.028	0.519 ± 0.037	0.458 ± 0.051
	Cefotaxime	AUROC	0.791 ± 0.021	0.783 ± 0.016	0.795 ± 0.022	**0.800 ± 0.021**	0.790 ± 0.021	0.762 ± 0.017
		AUPRC	0.447 ± 0.041	0.461 ± 0.028	0.467 ± 0.046	0.496 ± 0.046	**0.504 ± 0.040**	0.419 ± 0.050
	Trimethoprim-sulfamethoxazole	AUROC	0.679 ± 0.041	0.700 ± 0.019	0.702 ± 0.015	**0.704 ± 0.023**	0.686 ± 0.016	0.655 ± 0.039
		AUPRC	0.427 ± 0.043	**0.453 ± 0.022**	0.452 ± 0.033	0.452 ± 0.041	0.456 ± 0.028	0.406 ± 0.045
Klebsiella pneumoniae	Ciprofloxacin	AUROC	0.693 ± 0.032	0.700 ± 0.043	**0.720 ± 0.036**	0.715 ± 0.043	0.695 ± 0.039	0.693 ± 0.029
		AUPRC	0.279 ± 0.064	0.265 ± 0.051	**0.308 ± 0.072**	0.287 ± 0.046	0.259 ± 0.057	0.264 ± 0.059
	Cefotaxime	AUROC	0.730 ± 0.040	0.708 ± 0.025	**0.746 ± 0.042**	0.723 ± 0.040	0.734 ± 0.038	0.737 ± 0.031
		AUPRC	0.366 ± 0.075	0.351 ± 0.067	0.420 ± 0.091	0.367 ± 0.087	0.393 ± 0.079	**0.426 ± 0.066**
	Trimethoprim-sulfamethoxazole	AUROC	0.654 ± 0.030	**0.688 ± 0.028**	0.676 ± 0.017	0.657 ± 0.041	0.664 ± 0.035	0.662 ± 0.045
		AUPRC	0.235 ± 0.043	**0.264 ± 0.023**	0.250 ± 0.033	0.240 ± 0.045	0.231 ± 0.034	0.239 ± 0.036
	Piperacillin-tazobactam	AUROC	0.782 ± 0.028	0.796 ± 0.024	**0.810 ± 0.022**	0.805 ± 0.018	0.754 ± 0.033	0.792 ± 0.024
		AUPRC	0.625 ± 0.072	0.653 ± 0.067	**0.675 ± 0.069**	0.666 ± 0.061	0.616 ± 0.083	0.628 ± 0.070
Staphylococcus aureus	Inducible clindamycin resistance	AUROC	0.787 ± 0.025	0.787 ± 0.024	**0.804 ± 0.023**	0.799 ± 0.029	0.773 ± 0.022	0.797 ± 0.029
		AUPRC	0.437 ± 0.033	0.478 ± 0.043	0.475 ± 0.035	**0.510 ± 0.043**	0.452 ± 0.037	0.478 ± 0.049
	Oxacillin	AUROC	0.822 ± 0.023	**0.847 ± 0.021**	0.844 ± 0.021	0.836 ± 0.025	0.834 ± 0.025	0.833 ± 0.028
		AUPRC	0.598 ± 0.058	**0.647 ± 0.042**	0.619 ± 0.046	0.642 ± 0.040	0.628 ± 0.040	0.604 ± 0.060
	Benzylpenicillin	AUROC	0.799 ± 0.011	0.812 ± 0.011	0.819 ± 0.012	**0.820 ± 0.012**	0.728 ± 0.010	0.737 ± 0.040
		AUPRC	0.864 ± 0.011	0.882 ± 0.009	0.885 ± 0.011	**0.887 ± 0.009**	0.811 ± 0.010	0.813 ± 0.034

Results are reported as mean ± standard deviation over best models from 10 random train-test splits. Best performing models in terms of AUROC are marked in green for each species-antimicrobial combination. Combinations also studied by Weis et al. (16) are marked grey. Abbreviations: AUROC, area under the receiver operating characteristic curve; AUPRC, area under the precision-recall curve

Table S3: Overview of specimen sources included in the dataset

	Samples	
**Specimen source**	**[n]**	**%**
Urinary tract	5,951	39.7%
Respiratory tract	3,349	22.3%
Gastrointestinal tract	2,025	13.5%
Superficial skin	1,907	12.7%
Blood/aspirates	660	4.4%
Other	511	3.4%
Deep tissue	427	2.8%
Foreign body	118	0.8%
Missing source information	57	0.4%
Total	15,005	100.0%

Overview of the specimen types collected in 2023-2024 for *Escherichia coli* (n=7,897), *Klebsiella pneumoniae* (n=2,444) and *Staphylococcus aureus* (n=4,664).

Table S4: Resistance rates and sample sizes of datasets for the prospective observation of predictive performance

Species	Antimicrobial agent	Resistance rates [% (n/N)]				
		**Training Data 2023**	**Test Data 2023**	**Test Data 2024 Jan-Jun**	**Test Data 2024 Jul-Dec**	**Test Data 2025 Jan-Jun**
Escherichia coli	Ampicillin	51.5% (2,084/4,050)	52.3% (481/919)	52.3% (692/1,324)	53.4% (843/1,580)	54.7% (1187/2,172)
	Ciprofloxacin	18.2% (731/4,013)	16.9% (164/969)	15.2% (203/1,332)	18.5% (293/1,583)	19.2% (415/2,159)
	Cefotaxime	17.4% (696/3,997)	15.7% (154/979)	12.5% (166/1,328)	14.7% (231/1,575)	16.3% (352/2,154)
	Trimethoprim-sulfamethoxazole	27.9% (1,119/4,014)	27.1% (260/959)	30.9% (411/1,329)	26.5% (419/1,583)	30.3% (657/2,165)
Klebsiella pneumoniae	Ciprofloxacin	12.9% (160/1,238)	10.4% (33/317)	14.5% (54/373)	13.4% (69/516)	18.1% (113/625)
	Cefotaxime	16.3% (205/1,254)	15.0% (45/301)	17.4% (65/373)	16.1% (83/514)	19.7% (123/625)
	Trimethoprim-sulfamethoxazole	14.5% (183/1,258)	13.1% (39/298)	15.4% (57/371)	15.9% (82/516)	22.0% (138/627)
	Piperacillin-tazobactam	32.9% (417/1,268)	30.1% (83/276)	28.3% (104/367)	22.9% (118/516)	19.3% (120/623)
Staphylococcus aureus	Inducible clindamycin resistance	15.2% (341/2,250)	14.6% (81/554)	17.0% (145/851)	15.9% (137/864)	17.1% (242/1,416)
	Oxacillin	10.4% (240/2,310)	9.6% (55/570)	10.8% (94/872)	12.5% (112/896)	8.9% (129/1,455)
	Benzylpenicillin	60.2% (1,406/2,337)	60.6% (335/553)	59.9% (525/876)	68.4% (614/898)	60.5% (883/1,460)

Antimicrobial susceptibility test results were performed and interpreted following EUCAST guidelines and EUCAST clinical breakpoints. Sample sizes and resistance rates for 2023 test and training data are reported as the average over 10 test-train splits.

Table S5: Overview of antimicrobial susceptibility test results before binary labeling into “resistant” and “susceptible” (2023–2024)

Species	Antimicrobial agent	Samples [N]	Resistance label [% (n)]		
			Susceptible (S)	Susceptible, increased exposure (I)	Resistant (R)
Escherichia coli	Ampicillin	7,873	47.9% (3,773)	NA	52.1% (4,100)
	Cefotaxime	7,879	84.0% (6,619)	0.2% (13)	15.8% (1,247)
	Ciprofloxacin	7,897	77.1% (6,088)	5.3% (418)	17.6% (1,391)
	Trimethoprim-sulfamethoxazol	7,885	71.9% (5,669)	0.1% (8)	28.0% (2,208)
Klebsiella pneumoniae	Cefotaxime	2,442	82.9% (2,024)	0.8% (20)	16.3% (398)
	Ciprofloxacin	2,444	82.0% (2,004)	5.1% (124)	12.9% (316)
	Piperacillin-tazobactam	2,427	70.3% (1,705)	NA	29.7% (722)
	Trimethoprim-sulfamethoxazol	2,442	84.8% (2,070)	0.5% (12)	14.7% (360)
Staphylococcus aureus	Benzylpenicillin	4,664	38.3% (1,784)	NA	61.7% (2880)
	Inducible clindamycin resistance	4,519	84.4% (3,815)	NA	15.6% (704)
	Oxacillin	4,648	89.2% (4,147)	NA	10.8% (501)

Antimicrobial susceptibility test results were performed and interpreted following EUCAST guidelines and EUCAST clinical breakpoints. Abbreviations: NA, not applicable.

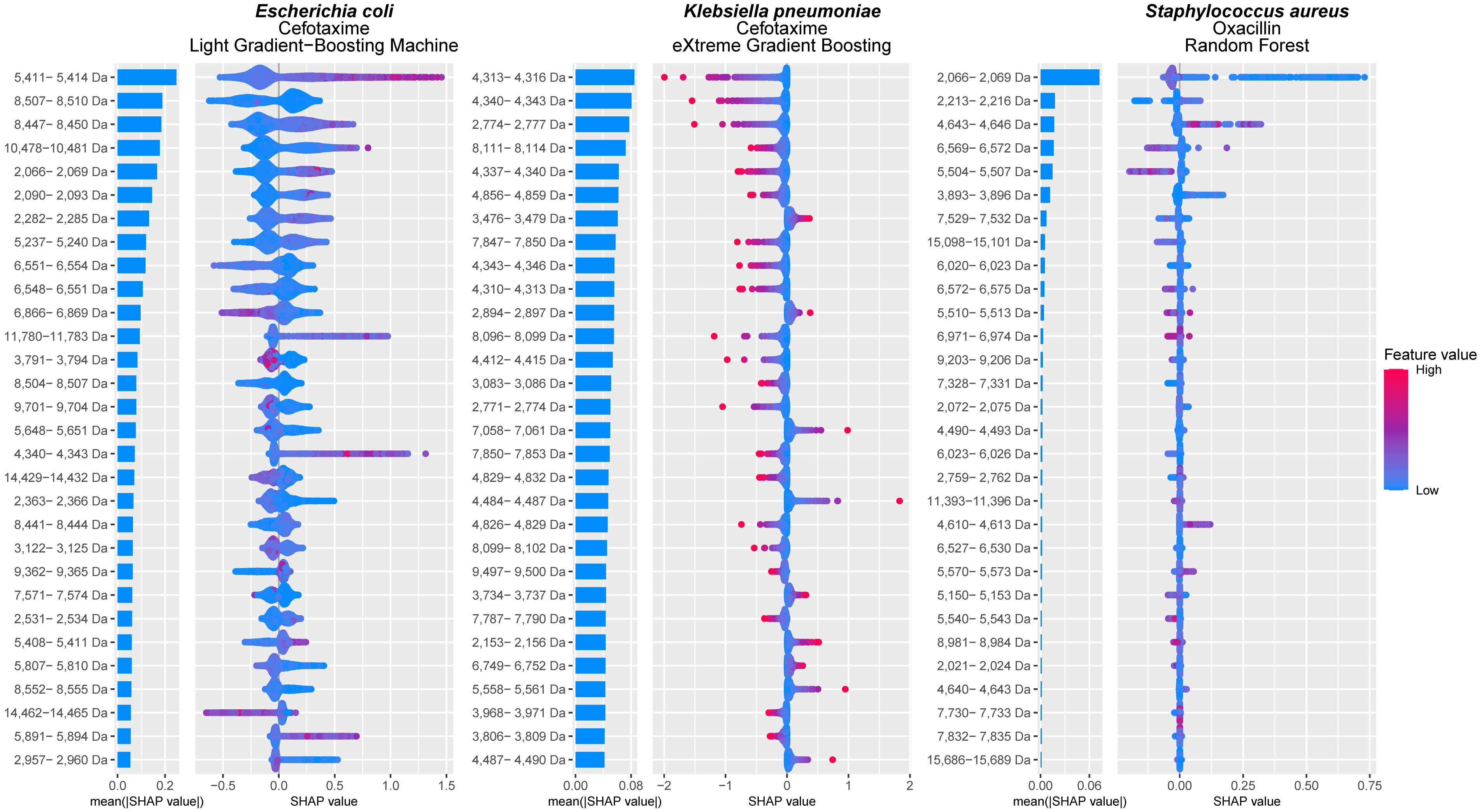

Figure S1: SHAP analysis of three selected machine learning models from our main benchmark (Figure 2). Features with the highest impact on the respective model prediction are displayed. Depicted on the left columns (for each species), the bar plots summarize the mean Shapley value, reflecting the features' average impact on the model output. The plots in the right columns visualize the Shapley value distributions over all samples, reflecting their contribution to the model output. The colors of each sample indicate the feature value (the intensity value in the respective bin of the mass spectrometry profile).

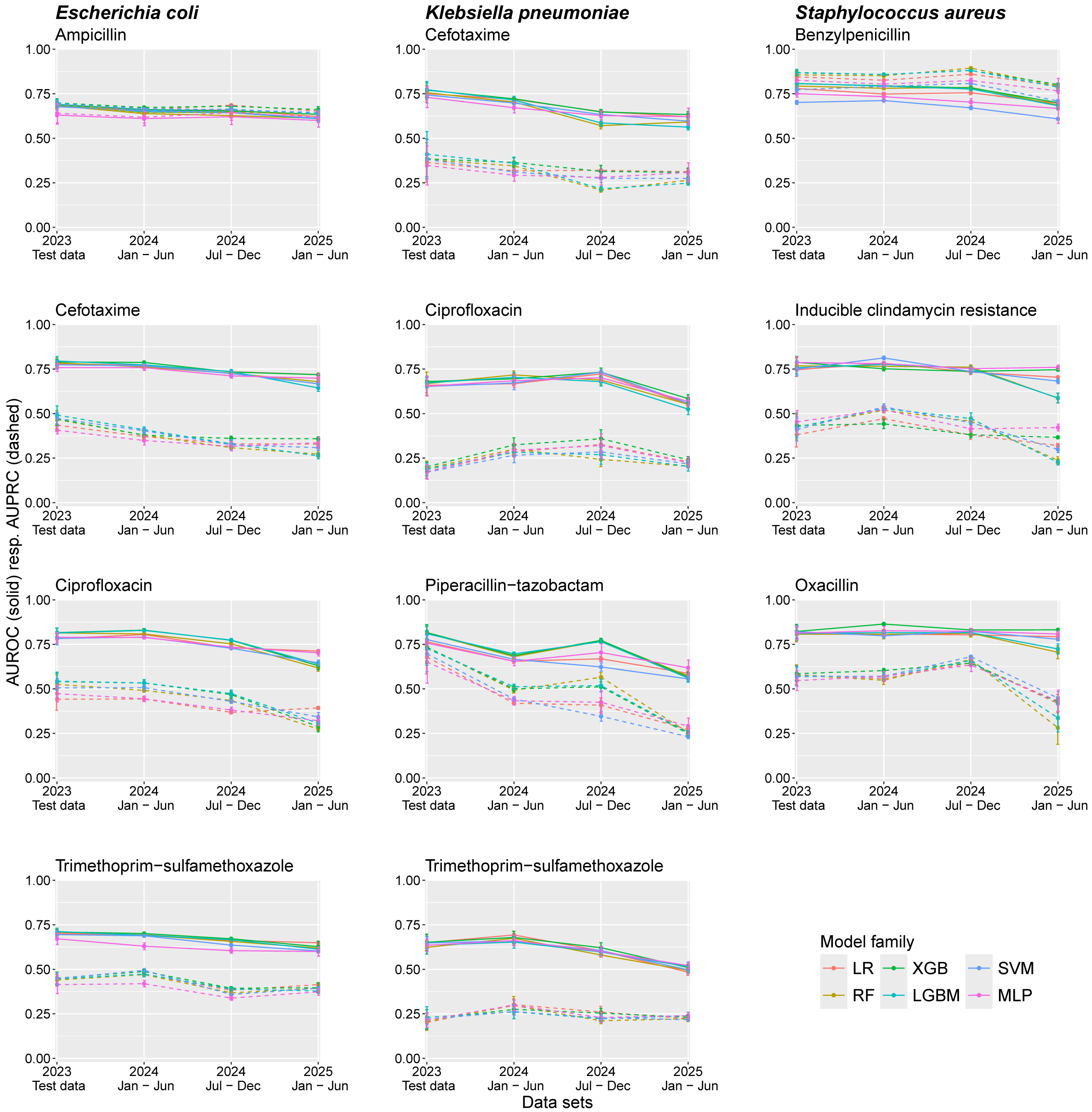

Figure S2: Performance of trained classifiers over time. Own study data were used for each species-antimicrobial combination, models are trained on data from Jan 2023-Dec 2023 and evaluated on prospective data from the following 3 half years. Model performance (averaged over best models from 10 random train-test splits, mean ± SD) in terms of area under the receiver operating characteristic curve (AUROC, solid lines) and area under the precision-recall curve (AUPRC, dashed lines). Abbreviations: LR, logistic regression; RF, random forest; XGB, eXtreme Gradient Boosting; LGBM, Light Gradient-Boosting Machine; SVM, support vector machine; MLP, multilayer perceptron

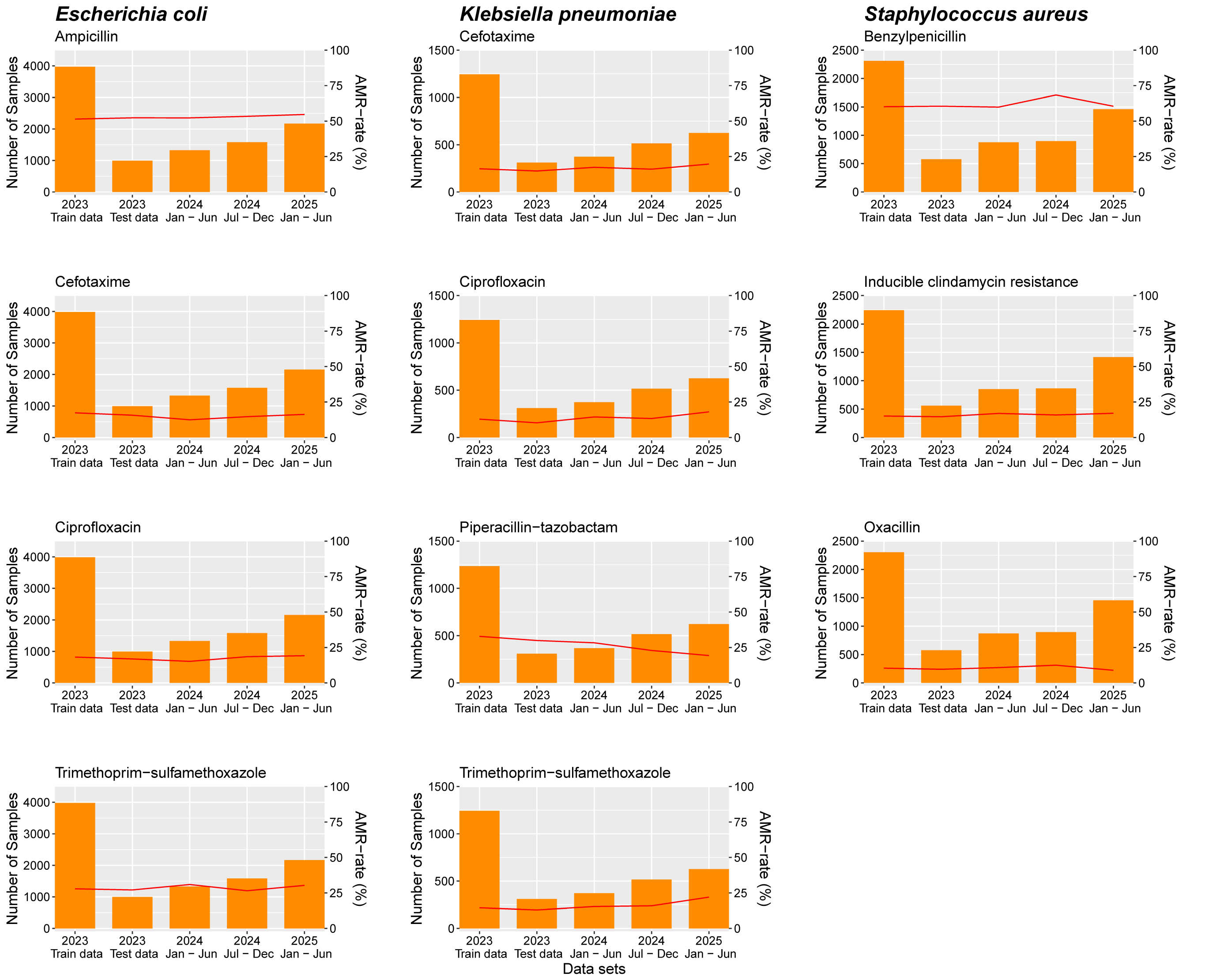

Figure S3: Datasets for temporal analysis of classifiers. Own study data were used for each species-antimicrobial combination. Models were trained on 2023 data and evaluated on prospective data from the following three half years. Sample sizes (orange bars) and resistance rates (red line) of the datasets used in the study of classifier performance are shown over time (Figure 4). Sample sizes and resistance rates for 2023 test and training data are reported as the average over 10 test-train splits.

References

1. Friedman J, Hastie T, Tibshirani R. 2010. Regularization Paths for Generalized Linear Models via Coordinate Descent. J Stat Softw 33:1-22.

2. Wright MN, Ziegler A. 2017. ranger: A Fast Implementation of Random Forests for High Dimensional Data in C plus plus and R. Journal of Statistical Software 77:1-17.

3. Chen T, Guestrin C. 2016. XGBoost: A Scalable Tree Boosting System, abstr Proceedings of the 22nd ACM SIGKDD International Conference on Knowledge Discovery and Data Mining, San Francisco, California, USA, Association for Computing Machinery,

4. Ke G, Meng Q, Finley T, Wang T, Chen W, Ma W, Ye Q, Liu T-Y. 2017. LightGBM: A Highly Efficient Gradient Boosting Decision Tree, abstr

5. Cortes C, Vapnik V. 1995. Support-Vector Networks. Machine Learning 20:273-297.

6. Gorishniy Y, Rubachev I, Khrulkov V, Babenko A. 2021. Revisiting deep learning models for tabular data, abstr Proceedings of the 35th International Conference on Neural Information Processing Systems,

7. Bengtsson H. 2024. future: Unified Parallel and Distributed Processing in R for Everyone, CRAN, https://cran.r-project.org/package=future.

8. Lang M, Binder M, Richter J, Schratz P, Pfisterer F, Coors S, Au Q, Casalicchio G, Kotthoff L, Bischl B. 2019. mlr3: A modern object-oriented machine learning framework in R. Journal of Open Source Software 4:1903.

9. Sonabend R, Schratz P, Fischer S. 2025. mlr3extralearners: Extra Learners For mlr3, https://github.com/mlr-org/mlr3extralearners.

10. Binder M, Pfisterer F, Lang M, Schneider L, Kotthoff L, Bischl B. 2021. mlr3pipelines - Flexible Machine Learning Pipelines in R. Journal of Machine Learning Research 22:1-7.

11. Hocking T. 2024. mlr3resampling: Resampling Algorithms for ‘mlr3’ Framework, CRAN, https://cran.r-project.org/package=mlr3resampling.

12. Becker M, Lang M, Richter J, Bischl B, Schalk D. 2024. mlr3tuning: Hyperparameter Optimization for ‘mlr3’, CRAN, https://cran.r-project.org/package=mlr3tuning.

13. Lang M, Schratz P, Becker M. 2025. mlr3verse: Easily Install and Load the 'mlr3' Package Family, CRAN, https://cran.r-project.org/package=mlr3verse.

14. Komisarczyk K, Kozminski P, Maksymiuk S, Lorenz AK, Spytek M, Krzyzinski M, Biecek P. 2025. treeshap: Compute SHAP Values for Your Tree-Based Models Using the 'TreeSHAP' Algorithm, CRAN, https://cran.r-project.org/package=treR package version 0.3.1eshap.

15. Mayer M. 2025. shapviz: SHAP Visualizations, CRAN, https://cran.r-project.org/package=shapviz.

16. Weis C, Cuénod A, Rieck B, Dubuis O, Graf S, Lang C, Oberle M, Brackmann M, Søgaard KK, Osthoff M, Borgwardt K, Egli A. 2022. Direct antimicrobial resistance prediction from clinical MALDI-TOF mass spectra using machine learning. Nat Med 28:164-174.
